# Clinical profile and predictors of adverse outcomes in pediatric dengue fever presenting with febrile illness: a prospective observational study in Burao, Somaliland

**DOI:** 10.3389/fped.2026.1891591

**Published:** 2026-07-14

**Authors:** Mohamed Omer Farah, Dek Kahin Yosef

**Affiliations:** 1 School of Nursing and Midwifery, College of Health Science, Burao Central University, Burao, Somaliland, Somalia; 2 School of Postgraduate Studies and Research, University of Burao, Burao, Somaliland, Somalia

**Keywords:** abdominal pain, bleeding manifestations, dengue fever, fever duration, pediatric dengue

## Abstract

**Background:**

Dengue fever is a growing public health concern globally, and there is limited pediatric-focused data available in Somaliland. This study aimed to prospectively characterize the clinical profile and identify predictors of adverse outcomes among children presenting with febrile illness, suspected of dengue, in Burao, Somaliland.

**Methods:**

This prospective observational study was conducted between September 2025 and April 2026 in pediatric outpatient and inpatient departments of hospitals in Burao. Children aged 0–18 years with acute febrile illness were consecutively enrolled. Clinical assessments and dengue confirmation using rapid diagnostic tests were performed. Disease severity followed WHO 2009 guidelines. Descriptive statistics summarized demographic and clinical characteristics. Multinomial logistic regression, using a prespecified causal framework rather than univariable screening, identified factors associated with recovery, complications, and death. Adjusted relative risk ratios (RRRs) with 95% confidence intervals (CIs) were reported.

**Results:**

Of the 186 participants (mean age, 7.86 years; 61.8% male; 83.3% urban residents), common symptoms included rash (60.8%), nausea/vomiting (89.3%), abdominal pain (69.3%), bleeding manifestations (51.1%), and hepatomegaly (47.3%). Most children (83.9%) recovered, 12.4% developed complications, and 3.7% died. Bleeding manifestations were independently associated with a higher relative likelihood of complications than recovery (adjusted relative risk ratio [RRR] = 5.07, 95% CI: 1.42–18.05; *p* = 0.012). Longer fever duration was independently associated with a higher relative likelihood of death than recovery (RRR = 1.34, 95% CI: 1.07–1.68; *p* = 0.011). A higher maximum recorded temperature was associated with an increased relative likelihood of complications (RRR = 1.65, 95% CI: 1.01–2.68; *p* = 0.044). Abdominal pain was associated with a lower relative likelihood of complications (RRR = 0.28, 95% CI: 0.08–1.00; *p* = 0.049). Age, sex, and residence were not independently associated with the outcomes.

**Conclusion:**

Pediatric dengue in Burao has favorable outcomes, but some children develop complications or die. Bleeding manifestations and prolonged fever independently predict adverse outcomes, while higher temperature and abdominal pain have borderline associations. Age, sex, and residence do not predict prognosis. Early recognition of bleeding signs and prolonged fever can improve triage, management, and outcomes. Prospective studies should validate these predictors for dengue care in Somaliland.

## Graphical Abstract

**Figure:**
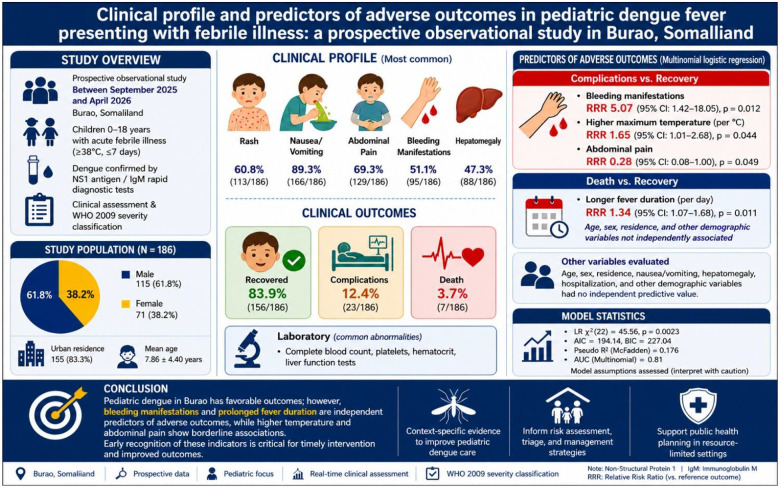
Visual abstract summarizing the clinical profile and predictors of adverse outcomes in pediatric dengue fever cases in Burao, Somaliland. This study highlights the predominant clinical features and favorable outcomes in most patients and identifies bleeding manifestations and prolonged fever as key predictors of adverse outcomes in patients with dengue fever.

## Highlights

- There are limited pediatric-specific data on dengue fever in Somaliland, necessitating a prospective study to characterize the clinical profiles and identify predictors of adverse outcomes in children with febrile illness.
- The study found that most children recovered; however, bleeding manifestations and prolonged fever duration were independently associated with an increased risk of complications and mortality. Additionally, a higher maximum recorded temperature was linked to a greater likelihood of complications, whereas abdominal pain showed a borderline association with a lower risk of complications.
- Early recognition of these clinical indicators is crucial for timely intervention and informing targeted public health strategies to improve pediatric dengue management in resource-limited urban settings in Burao, Somaliland.

## Background

Dengue virus (DENV) represents a significant global public health challenge, with an estimated 390 million annual infections, primarily transmitted by *Aedes* mosquitoes (1). The clinical spectrum of dengue ranges from asymptomatic or subclinical infection to severe disease, with asymptomatic or subclinical infections estimated to account for 40%–80% of pediatric cases (2, 3). Symptomatic infections commonly present as acute febrile illnesses and may progress to severe complications, including dengue hemorrhagic fever (DHF) and dengue shock syndrome (DSS) (4, 5). The burden of dengue has been increasing worldwide, including in Africa, where limited epidemiological data have hindered a comprehensive understanding of the disease (6). Factors such as rapid urbanization, climate variability, and increased human mobility contribute to the expansion of dengue transmission, highlighting the importance of generating context-specific evidence to inform public health strategies (7–9).

In Somaliland, studies on dengue have predominantly focused on adult populations, providing preliminary insights into seroprevalence, clinical manifestations, and seasonal trends. Retrospective analyses in public hospitals have identified dengue incidence among suspected cases, revealing urban predominance, adult male susceptibility, and a range of clinical features, including fever, gastrointestinal symptoms, myalgia, and neurological involvement (10). However, these studies have several limitations, including reliance on retrospective data, restricted sample populations, and limited focus on pediatric patients. Consequently, there is a critical knowledge gap regarding the clinical profile, disease severity, and early warning signs of dengue in children, who are particularly vulnerable to severe manifestations owing to physiological differences and immune responses (1, 11, 12).

The lack of prospective, pediatric-focused data impedes accurate clinical recognition and timely intervention, particularly in settings where other febrile illnesses, such as malaria, bacterial infections, and viral gastroenteritis, are endemic and present with overlapping symptoms (13). This diagnostic challenge may delay appropriate management and increase the risk of complications and hospitalization (14). Furthermore, limited understanding of pediatric disease patterns hinders public health planning, including targeted vector control, community education, and resource allocation, to effectively mitigate outbreaks (15).

Therefore, this study was designed to address these critical gaps by prospectively assessing the clinical profile and predictors of adverse outcomes among pediatric patients presenting with dengue fever in the context of febrile illness in Burao, Somaliland. By generating systematically collected, context-specific evidence, the study aims to improve early clinical recognition, support timely risk stratification, and inform targeted public health interventions for pediatric populations. Ultimately, the findings are expected to contribute to reducing dengue-related morbidity and mortality among children and strengthen the regional capacity for effective dengue surveillance and response, while complementing existing adult-focused evidence to provide a more comprehensive understanding of dengue epidemiology in Somaliland.

## Methodology

### Study design and setting

This prospective observational study was conducted at the pediatric outpatient and inpatient departments of selected hospitals in Burao, Somaliland, between September 2025 and April 2026. Burao, the largest city in the Togdheer region, has a semi-arid climate with distinct rainy and dry seasons that influence mosquito breeding and arboviral transmission. This study aimed to capture real-time clinical data on pediatric dengue cases in an urban environment.

### Study population, eligibility, and sample size determination

The study population included children aged 0–18 years with acute febrile illnesses, defined as a body temperature of ≥38 °C within the previous 7 days. Children with chronic illnesses, immunodeficiency disorders, and incomplete clinical data were excluded. The sample size was determined using a single-population proportion formula, assuming a 12% dengue prevalence based on previous studies in Somaliland (10), a 95% confidence interval, and a 5% margin of error, with an additional 10% added for potential non-response bias. This resulted in a minimum sample size of 186. Eligible participants were consecutively recruited until the required sample size was achieved.

### Sampling technique

A consecutive sampling technique was employed, in which all children who met the inclusion criteria and presented to the pediatric departments during the study period were approached for enrollment. This method ensured that a representative sample of children presenting with febrile illnesses during the peak and off-peak dengue seasons in the study area was obtained.

### Data collection

#### Questionnaire tool

A structured questionnaire was used to collect sociodemographic data, including age, sex, place of residence, and history of febrile illnesses. Clinical information was obtained through direct patient assessments and parental interviews, focusing on dengue symptoms, such as fever patterns, gastrointestinal complaints, rash, myalgia, headache, respiratory symptoms, and neurological manifestations. Disease severity was categorized according to the 2009 World Health Organization (WHO) dengue guidelines as dengue without warning signs, dengue with warning signs, or severe dengue (16). While the WHO classification informed clinical assessment and severity stratification, the primary outcomes analyzed were recovery, development of complications, and death, reflecting direct clinical endpoints relevant to risk prediction.

### Laboratory methods

Laboratory confirmation of dengue infection was performed using rapid diagnostic tests (RDTs) for NS1 antigen and IgM antibodies. Blood samples were collected aseptically and processed in the hospital laboratory within 1 hour of collection. Hematological investigations, including complete blood count, platelet count, hematocrit, and liver function tests, were conducted to assess disease severity and monitor complications. The study population consisted of 186 participants, comprising both laboratory-confirmed and clinically suspected cases without laboratory confirmation. Due to resource constraints, molecular confirmation and systematic exclusion of other acute febrile illnesses, such as malaria, bacterial infections, and viral gastroenteritis, were not performed. Clinical assessment included evaluation for alternative diagnoses, and patients with strong clinical suspicion of dengue despite negative RDT results were closely monitored for disease progression. Consequently, data on co-infections were not collected, limiting the ability to assess their frequency or impact on disease severity and clinical outcomes.

### Data management and statistical analysis

Data collected from questionnaires and laboratory records were entered into Microsoft Excel 2021 and checked for completeness, consistency, and range errors prior to analysis. The cleaned dataset was exported to Stata version 17 (StataCorp, College Station, TX, USA) for statistical analysis. Descriptive statistics were used to summarize sociodemographic characteristics, clinical features, and laboratory findings. Normally distributed continuous variables were expressed as mean ± standard deviation, and non-normally distributed variables as median with interquartile range (IQR). Categorical variables were presented as frequencies and percentages.

Multinomial logistic regression models were prespecified using a causal framework incorporating demographic variables (age, sex, and residence) and clinically relevant baseline features (fever duration, maximum temperature, nausea/vomiting, abdominal pain, bleeding manifestations, and hepatomegaly). Variable selection was not based on univariable *p*-value screening. The outcome variable was clinical outcome categorized as recovered (reference), complications, and death. Model assumptions were evaluated by assessing multicollinearity among the predictor variables using variance inflation factors (mean VIF = 1.25; range: 1.06–1.58), indicating no evidence of problematic multicollinearity. Model fit was assessed using Akaike’s information criterion (AIC = 195.71), Bayesian information criterion (BIC = 259.79), and the log-likelihood (−77.85). Overall model significance was evaluated using the likelihood ratio test (LR *χ*²(18) = 35.99, *p* = 0.0071).

Sensitivity analyses were conducted to evaluate the robustness of the primary multinomial logistic regression model. Alternative model specifications included (1) adding rash as an additional predictor, (2) replacing continuous age with categorical age groups, and (3) including hospitalization as an additional covariate. The consistency of effect estimates and overall study conclusions across these alternative specifications was compared with that of the prespecified primary model. The prespecified model was retained as the primary analysis unless alternative specifications materially changed the estimated associations.

## Results

### Socio-demographic and clinical characteristics

A total of 186 participants were included in the study, comprising both laboratory-confirmed and clinically suspected dengue cases without laboratory confirmation of the diagnosis. This number represents all eligible children presenting with acute febrile illness suspected of dengue to the pediatric outpatient and inpatient departments during the study period. The mean age was 7.86 ± 4.40 years, with a median age of 8 years (IQR: 4–12 years). Males accounted for 115 (61.8%) of the study population, while females comprised 71 (38.2%) of the study population. Most participants resided in urban areas (155, 83.3%), with 31 (16.7%) residing in rural settings. Regarding age distribution, 74 (39.8%) were aged 1–5 years, 53 (28.5%) were 6–11 years old, 57 (30.6%) were 12–16 years old, and 2 (1.1%) were > 17 years old. The clinical features were common in the cohort. Rash was present in 113 (60.8%) patients, nausea/vomiting in 166 (89.3%), abdominal pain in 129 (69.3%), bleeding manifestations in 95 (51.1%), and hepatomegaly in 88 (47.3%) patients (Table 1). Hospitalization was recorded in 135 (72.6%) patients (Table 1).

**Table 1:** Socio-demographic, and clinical characteristics (*N* = 186).

Variable	Category	*n* (%) or Mean ± SD
Age (years)	Mean ± SD	7.86 ± 4.40
	Median (IQR)	8 (4–12)
Sex	Male	115 (61.8)
	Female	71 (38.2)
Residence	Urban	155 (83.3)
	Rural	31 (16.7)
Age group	1–5 years	74 (39.8)
	6–11 years	53 (28.5)
	12–16 years	57 (30.6)
	>17 years	2 (1.1)
Clinical features		
Rash	Yes	113 (60.8)
Nausea/vomiting	Yes	166 (89.3)
Abdominal pain	Yes	129 (69.3)
Bleeding manifestations	Yes	95 (51.1)
Hepatomegaly	Yes	88 (47.3)
Hospitalized	Yes	135 (72.6)

### Clinical outcomes according to demographic and clinical characteristics (*N* = 186)

Among the 186 pediatric patients with dengue, 156 (83.9%) recovered, 23 (12.4%) developed complications, and 7 (3.7%) died (Table 2). Recovery was the most frequent outcome in all demographic and clinical subgroups. Among male patients, 88.7% recovered, compared to 76.1% of female patients. Recovery was more common among urban (85.2%) than rural residents (77.4%). Patients presenting with rash, bleeding manifestations, or abdominal pain had a higher proportion of complications and deaths than those without these clinical features. The median duration of fever before presentation was 4 (IQR: 3–5) days among patients who recovered, 5 (IQR: 2–8) days among those who developed complications, and 5 (IQR: 5–12) days among those who died. Patients with complications had a slightly higher median maximum recorded temperature (39 [38.5–40] °C) than those who recovered (39 [38–39] °C), whereas patients who died had a lower median maximum temperature (38.4 [37.8–38.9] °C). The median hospital stay increased across outcome categories, from 3 (IQR: 3–5) days among recovered patients to 4 (IQR: 2–10.5) days among those with complications and 6 (IQR: 5–8) days among those who died (Table 2).

**Table 2:** Distribution of clinical outcomes by demographic and clinical characteristics (*N* = 186).

Variable	Category	Recovered *n* (%)	Complications *n* (%)	Death *n* (%)
Clinical outcome	Overall	156 (83.9)	23 (12.4)	7 (3.7)
Sex	Male	102 (88.7)	9 (7.8)	4 (3.5)
	Female	54 (76.1)	14 (19.7)	3 (4.2)
Residence	Urban	132 (85.2)	17 (11.0)	6 (3.9)
	Rural	24 (77.4)	6 (19.4)	1 (3.2)
Rash	Yes	90 (79.6)	16 (14.2)	7 (6.2)
	No	66 (90.4)	7 (9.6)	0 (0.0)
Nausea/vomiting	Yes	141 (84.9)	19 (11.4)	6 (3.6)
	No	15 (75.0)	4 (20.0)	1 (5.0)
Abdominal pain	Yes	113 (87.6)	12 (9.3)	4 (3.1)
	No	43 (75.4)	11 (19.3)	3 (5.3)
Bleeding manifestations	Yes	75 (78.9)	15 (15.8)	5 (5.3)
	No	81 (89.0)	8 (8.8)	2 (2.2)
Hepatomegaly	Yes	76 (86.4)	9 (10.2)	3 (3.4)
	No	80 (81.6)	14 (14.3)	4 (4.1)
Hospitalization	Yes	116 (85.9)	16 (11.9)	3 (2.2)
	No	40 (78.4)	7 (13.7)	4 (7.8)
Age (years)	Median (IQR)	8 (4.5–12)	7 (3–12)	8 (2–12)
Fever duration (days)	Median (IQR)	4 (3–5)	5 (2–8)	5 (5–12)
Maximum temperature (°C)	Median (IQR)	39.0 (38–39)	39.0 (38.5–40)	38.4 (37.8–38.9)
Hospital stay (days)	Median (IQR)	3 (3–5)	4 (2–10.5)	6 (5–8)

### Multinomial logistic regression analysis

After adjustment for age, sex, residence, fever duration, maximum temperature, nausea/vomiting, abdominal pain, bleeding manifestations, and hepatomegaly. Bleeding manifestations were associated with a higher relative risk of developing complications compared to recovery (adjusted RRR = 5.07, 95% CI: 1.42–18.05). Higher maximum recorded temperature was associated with an increased relative risk of complications (adjusted RRR = 1.65, 95% CI: 1.01–2.68). Abdominal pain was associated with a lower relative risk of complications compared with recovery (adjusted RRR = 0.28, 95% CI: 0.08–1.00). Longer fever duration before presentation was associated with a higher relative risk of death compared with recovery (adjusted RRR = 1.34, 95% CI: 1.07–1.68). No independent associations were observed for sex, residence, age, nausea/vomiting, or hepatomegaly after adjustment (Table 3). Sensitivity analyses demonstrated that the primary findings were robust to alternative model specifications. Models including rash as an additional predictor and replacing continuous age with categorical age groups produced unstable estimates for the death outcome because of the small number of deaths (*n* = 7), but the overall conclusions remained unchanged. Inclusion of hospitalization as an additional covariate yielded effect estimates that were very similar to those of the primary model. Accordingly, the prespecified causal model was retained as the primary analysis (Supplementary Table S1).

**Table 3:** Multinomial logistic regression analysis of factors associated with clinical outcome (reference outcome = recovered).

Variable	Category	Complications vs. Recovered Adjusted RRR (95% CI)	*p*-value	Death vs. Recovered Adjusted RRR (95% CI)	*p*-value
Sex	Female (Ref)	1.00	–	1.00	–
	Male	0.37 (0.12–1.12)	0.078	1.27 (0.21–7.61)	0.797
Residence	Rural (Ref)	1.00	–	1.00	–
	Urban	0.57 (0.17–1.89)	0.361	1.70 (0.16–17.79)	0.658
Age (years)	Continuous	1.03 (0.91–1.17)	0.606	1.01 (0.83–1.25)	0.889
Fever duration (days)	Continuous	1.15 (0.98–1.35)	0.088	1.34 (1.07–1.68)	0.011
Maximum temperature (°C)	Continuous	1.65 (1.01–2.68)	0.044	0.68 (0.33–1.41)	0.303
Nausea/vomiting	No (Ref)	1.00	–	1.00	–
	Yes	0.46 (0.10–2.10)	0.319	0.46 (0.03–6.47)	0.566
Abdominal pain	No (Ref)	1.00	–	1.00	–
	Yes	0.28 (0.08–1.00)	0.049	0.24 (0.03–1.82)	0.168
Bleeding manifestations	No (Ref)	1.00	–	1.00	–
	Yes	5.07 (1.42–18.05)	0.012	5.59 (0.72–43.66)	0.101
Hepatomegaly	No (Ref)	1.00	–	1.00	–
	Yes	0.52 (0.16–1.69)	0.274	0.42 (0.07–2.58)	0.350

✓ Outcome variable: Clinical outcome (recovered, complications, death).

✓ Reference outcome: Recovered.

✓ Relative risk ratios (RRRs) were adjusted for age, sex, residence, fever duration, maximum temperature, nausea/vomiting, abdominal pain, bleeding manifestations, and hepatomegaly.

## Discussion

This prospective observational study provides one of the first pediatric-focused clinical characterizations of dengue fever in Somaliland, integrating clinical, demographic, and multivariate analyses of the data. The findings showed that most children recovered (83.9%), whereas a smaller proportion developed complications (12.4%) or died (3.7%) after hospitalization. Although most cases were uncomplicated, the presence of severe outcomes highlights the important clinical heterogeneity of pediatric dengue and reinforces the need for early identification of high-risk presentations in resource-limited settings. Bleeding manifestations and prolonged fever duration were the most consistent predictors of adverse outcomes, whereas demographic variables showed limited independent predictive value.

The study population was predominantly young children, with a male and urban predominance. This pattern is consistent with the findings from dengue-endemic regions, where younger age groups are more frequently affected owing to limited prior immunity (17). The urban clustering of cases reflects the ecological suitability of Aedes mosquito breeding in densely populated environments with inadequate water storage and waste management systems (18). Similar urban-driven transmission patterns have been reported across East Africa and Asia, where anthropogenic environmental conditions sustain vector proliferation despite semi-arid climates (19, 20). In Burao, these findings highlight the role of peri-domestic water storage practices and urban infrastructure limitations in maintaining transmission risk.

Clinically, fever was universal, and gastrointestinal symptoms were highly prevalent, including nausea/vomiting (89.3%) and abdominal pain (69.3%). These findings align with the known pediatric dengue phenotype, which is characterized by a systemic inflammatory response and capillary leakage. The relatively high frequency of bleeding manifestations and hepatomegaly suggests a substantial proportion of clinically significant disease (21, 22). Similar symptom distributions have been reported in pediatric cohorts from Southeast Asia (22–24), although variations are expected depending on circulating serotypes, host immunity, and healthcare access in the region. In the present study, the frequency of hemorrhagic features may also reflect delayed presentation and diagnostic overlap with other endemic febrile illnesses, such as malaria and bacterial infections. The diagnostic overlap between dengue and other endemic febrile illnesses, including malaria, bacterial infections, and viral gastroenteritis, presents a significant clinical challenge in resource-limited settings, such as Somaliland. Although systematic laboratory exclusion of these conditions was not feasible in this study, clinical assessments aimed to consider differential diagnoses. This limitation may have contributed to the delayed recognition and management of dengue cases, underscoring the need to strengthen diagnostic capacity for comprehensive febrile illness workups in similar contexts.

Most children recovered without complications, consistent with the self-limiting nature of primary dengue infections in the pediatric population. However, the observed complication and mortality rates remain clinically important and comparable to those reported from other low-and middle-income settings, where delayed diagnosis and limited supportive care capacity contribute to worse outcomes (25, 26). In contrast to high-resource settings, where mortality is rare, the presence of pediatric deaths in this cohort underscores the ongoing gaps in early recognition and access to critical care (27).

The observed association between bleeding manifestations and complications is consistent with dengue pathophysiology, in which vascular injury and thrombocytopenia contribute to hemorrhagic progression (28). Similar associations have been reported in studies from Thailand, India, and parts of Africa, where bleeding signs are recognized as important clinical warning indicators (29–32). The estimated adjusted relative risk suggests that patients with bleeding manifestations may have a substantially greater likelihood of developing complications than those without bleeding. However, the relatively wide confidence interval indicates uncertainty in the magnitude of the association, likely reflecting the limited number of complications.

Longer fever duration before presentation was associated with an increased relative risk of death, with each additional day of fever corresponding to an estimated 34% increase in the relative risk of death. This finding suggests that prolonged febrile illness may indicate delayed presentation or more advanced disease and highlights the importance of early clinical evaluation and management. Similar observations have been reported in pediatric cohorts from Latin America and Southeast Asia, where prolonged fever has been associated with the progression to severe dengue manifestations (33–35). Nevertheless, this estimate should be interpreted in the context of the small number of deaths observed in this study population.

A higher maximum recorded temperature was associated with an increased relative risk of complications. This finding is consistent with previous pediatric dengue studies, in which higher fever intensity has been associated with more severe disease manifestations, reflecting heightened systemic inflammatory responses (36–38). Elevated temperature may correspond to increased viral replication and immune activation, contributing to vascular permeability and plasma leakage (39–41). However, because the confidence interval was close to the null value and the statistical evidence was borderline, this finding should be interpreted cautiously and requires confirmation in larger studies. Temperature should therefore be considered alongside other clinical indicators rather than as an independent predictor of disease severity. Similar associations have been reported in pediatric cohorts from Southeast Asia and Latin America (42, 43).

Abdominal pain was associated with a lower relative risk of complications than recovery. This finding is unexpected because the World Health Organization recognizes abdominal pain as a warning sign for severe dengue, which is generally associated with plasma leakage and disease progression (1). The observed inverse association may reflect local case-mix variation, earlier healthcare-seeking behavior among patients with abdominal pain, or residual confounding factors, such as socioeconomic status. Furthermore, the confidence interval extended to the null value, and the statistical evidence was borderline; therefore, this finding should be interpreted cautiously and confirmed in larger prospective studies with larger sample sizes.

Although several adjusted associations were identified, some estimates, particularly those for maximum temperature and abdominal pain, were supported by statistical evidence close to the conventional threshold for significance. Given that multiple predictors were evaluated within a single multinomial regression model, these findings may have occurred by chance and should not be overinterpreted. Greater emphasis should therefore be placed on the magnitude and precision of the adjusted relative risk ratios and their potential clinical relevance rather than on p values alone. Further studies with larger sample sizes are needed to confirm these associations.

Demographic variables, including age, sex, and residence, were not independent predictors of outcome. This suggests that disease progression in pediatric dengue is primarily driven by clinical and biological responses rather than baseline demographic characteristics. Although females comprised only 38.2% of the study population, they exhibited more than twice the rate of complications compared to males. This discrepancy may reflect a combination of factors, including potential biological differences in immune response or hormonal influences that could affect disease progression. Behavioral factors, such as delayed healthcare-seeking among females or differential exposure risks, may also contribute. However, it is important to note that sex was not an independent predictor of adverse outcomes in the multivariable analysis, and the observed difference did not reach statistical significance. These findings suggest that while sex differences in complication rates may exist, larger studies are needed to clarify underlying mechanisms and confirm whether female sex constitutes a true risk factor for complications in pediatric dengue. Urban residence also showed no independent effect, suggesting that once infection occurs, severity is determined more by host–pathogen interactions than exposure setting.

From a clinical perspective, these findings suggest that bleeding manifestations, prolonged fever duration, higher maximum recorded temperature, and abdominal pain may contribute to the early clinical assessment of pediatric dengue, although the associations between maximum temperature and abdominal pain and dengue should be interpreted cautiously because of their borderline statistical evidence and the number of predictors evaluated in the multivariable model. Therefore, clinical decisions should consider these factors alongside the overall clinical presentation rather than in isolation. In resource-limited settings such as Somaliland, where dengue clinically overlaps with malaria and other febrile illnesses, strengthening triage systems and improving early diagnostic capacity may facilitate the timely recognition and management of children at an increased risk of adverse outcomes. From a public health perspective, the predominance of urban cases underscores the need for targeted vector control interventions, focusing on improved household water storage, environmental sanitation, and community education to reduce *Aedes* breeding sites.

Although the WHO 2009 dengue classification was used to categorize disease severity during clinical evaluation, our analysis focused on the clinical outcomes of recovery, complications, and mortality to provide clear, actionable endpoints for pediatric dengue management. This approach complements the WHO classification by linking clinical severity categories to concrete patient outcomes, aiding the early identification of children at risk for adverse events in resource-limited settings.

This study has several limitations. First, the small number of deaths (*n* = 7) limited the precision of the mortality estimates, resulting in wide confidence intervals for some adjusted relative risk ratios. Second, dengue diagnosis relied on rapid diagnostic tests detecting NS1 antigen and/or IgM antibodies without molecular confirmation or serotyping. Consequently, misclassification of dengue infection and the inability to distinguish primary from secondary infections or circulating serotypes may have influenced the observed clinical profile and outcomes. Third, systematic testing for co-infections, such as malaria and bacterial infections, was not performed because of resource limitations, and these conditions may have contributed to the clinical presentation and disease severity. Fourth, consecutive recruitment of children presenting to healthcare facilities may have resulted in selection bias by underrepresenting milder community cases, thereby limiting the generalizability of the findings.

Although the multivariate model was specified using a prespecified causal framework rather than univariate screening, several predictors were evaluated simultaneously. Consequently, some associations, particularly those with statistical evidence close to the conventional significance threshold (e.g., maximum temperature and abdominal pain), may represent chance findings rather than true associations. Therefore, these results should be interpreted cautiously, with greater emphasis placed on the magnitude and precision of the adjusted relative risk ratios and their confidence intervals rather than on p-values alone. Larger multicenter studies with external validation are required to confirm these findings. Despite these limitations, the prospective study design, standardized clinical assessment, and use of a theory-driven multivariate modeling approach strengthen the internal validity of the findings and provide important evidence on pediatric dengue outcomes in a resource-limited setting.

Pediatric dengue in Burao is characterized by predominantly favorable outcomes; however, a clinically important subset of children developed complications or died from the disease. Bleeding manifestations and prolonged fever duration were associated with adverse clinical outcomes, whereas a higher maximum recorded temperature and abdominal pain were associated with clinical outcomes in the adjusted analysis. These findings provide context-specific evidence to inform clinical risk assessment, support improved triage and timely management, and guide public health strategies aimed at reducing the burden of severe dengue in children in Somaliland. Further studies with larger sample sizes are required to confirm these associations.

## Conclusions

This prospective study provides important pediatric-specific clinical insights into dengue fever in Burao, Somaliland, demonstrating that although most children recovered, a clinically important subset experienced complications or died. Bleeding manifestations and prolonged fever duration were associated with adverse clinical outcomes, whereas a higher maximum recorded temperature and abdominal pain were also associated with clinical outcomes in the adjusted analysis. These findings support the use of comprehensive clinical assessments to identify children at increased risk of adverse outcomes and highlight the need for strengthened diagnostic capacity, timely clinical management, and targeted public health interventions, particularly in urban settings where the transmission risk is higher. Larger prospective multicenter studies are needed to confirm these associations and further refine the risk stratification for pediatric dengue in resource-limited settings.

## Data Availability

The raw data supporting the conclusions of this article will be made available by the authors, without undue reservation.

## References

[1] World Health Organization. Dengue (2025). Available online at: https://www.who.int/news-room/fact-sheets/detail/dengue-and-severe-dengue (Accessed August 21, 2025).

[2] De Santis O Bouscaren N Flahault A. Asymptomatic dengue infection rate: a systematic literature review. Heliyon. (2023) 9(9):e20069. 10.1016/j.heliyon.2023.e20069 37809992 PMC10559824

[3] Centers for Disease Control and Prevention. Dengue (2025). Available online at: https://www.cdc.gov/yellow-book/hcp/travel-associated-infections-diseases/dengue.html (Accessed April 23, 2025).

[4] Kularatne SA Dalugama C. Dengue infection: global importance, immunopathology and management. Clin Med (Lond). (2022) 22(1):9–13. 10.7861/clinmed.2021-0791 35078789 PMC8813012

[5] Kalayanarooj S. Clinical manifestations and management of dengue/DHF/DSS. Trop Med Health. (2011) 39(4 Suppl):83–87. 10.2149/tmh.2011-S10 22500140 PMC3317599

[6] Gainor EM Harris E LaBeaud AD. Uncovering the burden of dengue in Africa: considerations on magnitude, misdiagnosis, and ancestry. Viruses. (2022) 14(2):233. 10.3390/v14020233 35215827 PMC8877195

[7] e Silva LMA Bastos CS. The role of weather and city environment in dengue outbreaks: what do we know so far? Obs Econ Latinoam. (2025) 23(1):e8564–e8564. 10.55905/oelv23n1-055

[8] Nikookar SH Hoseini S Dehghan O Fazelidinan M Enayati A. Dengue fever resurgence in Iran: an integrative review of causative factors and control strategies. Trop Med Infect Dis. (2025) 10(11):309. 10.3390/tropicalmed10110309 41295574 PMC12656587

[9] Gibb R Colón-González FJ Lan PT Huong PT Nam VS Duoc VT. Interactions between climate change, urban infrastructure and mobility are driving dengue emergence in Vietnam. Nat Commun. (2023) 14(1):8179. 10.1038/s41467-023-43954-0 38081831 PMC10713571

[10] Yosef DK Ismail AS Awil BS Hassan HA Hassan MA. Epidemiology of dengue fever in Somaliland: clinical features, and serological patterns from a retrospective study. BMC Infect Dis. (2025) 25(1):179. 10.1186/s12879-025-10558-6 39910527 PMC11800544

[11] Sami CA Tasnim R Hassan SS Khan AH Yasmin R Monir-Uz-Zaman M. Clinical profile and early severity predictors of dengue fever: current trends for the deadliest dengue infection in Bangladesh in 2022. IJID Reg. (2023) 9:42–48. 10.1016/j.ijregi.2023.09.001 37859805 PMC10582778

[12] Salazar Flórez JE Marín Velasquez K Segura Cardona ÁM Restrepo Jaramillo BN Ortega Díaz YE Giraldo Cardona LS. Clinical manifestations of dengue in children and adults in a hyperendemic region of Colombia. Am J Trop Med Hyg. (2024) 110(5):971–978. 10.4269/ajtmh.23-0717 38507814 PMC11066339

[13] Iroh Tam PY Obaro SK Storch G. Challenges in the etiology and diagnosis of acute febrile illness in children in low- and middle-income countries. J Pediatric Infect Dis Soc. (2016) 5(2):190–205. 10.1093/jpids/piw016 27059657 PMC7107506

[14] Mallhi TH Khan AH Sarriff A Adnan AS Khan YH. Patients related diagnostic delay in dengue: an important cause of morbidity and mortality. Clin Epidemiol Glob Health. (2016) 4(4):200–201. 10.1016/j.cegh.2016.08.002

[15] Lessa CLS Fiuza BSD Hodel KVS Minafra C de Souza Gonçalves M Machado BAS. Understanding dengue underreporting: an analysis of the impacts for the world, Latin America and Brazil. Scientific World Journal. (2025) 2025:9993911. 10.1155/tswj/9993911 40704162 PMC12286677

[16] Hadinegoro SR. The revised WHO dengue case classification: does the system need to be modified? Paediatr Int Child Health. (2012) 32 Suppl 1(s1):33–38. 10.1179/2046904712z.00000000052 22668448 PMC3381438

[17] Kumar M Verma RK Nirjhar S Singh M. Dengue in children and young adults, a cross-sectional study from the western part of Uttar Pradesh. J Family Med Prim Care. (2020) 9(1):293–297. 10.4103/jfmpc.jfmpc_770_19 32110607 PMC7014909

[18] Kolimenakis A Heinz S Wilson ML Winkler V Yakob L Michaelakis A. The role of urbanisation in the spread of Aedes mosquitoes and the diseases they transmit-a systematic review. PLoS Negl Trop Dis. (2021) 15(9):e0009631. 10.1371/journal.pntd.0009631 34499653 PMC8428665

[19] Eder M Cortes F Teixeira de Siqueira Filha N Araújo de França GV Degroote S Braga C. Scoping review on vector-borne diseases in urban areas: transmission dynamics, vectorial capacity and co-infection. Infect Dis Poverty. (2018) 7(1):90. 10.1186/s40249-018-0475-7 30173661 PMC6120094

[20] Li J Lee M-C Jiang A-L Yan G Hsu K. Predicting environmental suitability and future spread range of An. stephensi in the greater horn of Africa using remote sensing and ensemble modeling. Int J Appl Earth Obs Geoinf. (2026) 146:105026. 10.1016/j.jag.2025.105026 41767644 PMC12948167

[21] Pothapregada S Kamalakannan B Thulasingham M Sampath S. Clinically profiling pediatric patients with dengue. J Glob Infect Dis. (2016) 8(3):115–120. 10.4103/0974-777x.188596 27621562 PMC4997795

[22] Jagadishkumar K Jain P Manjunath VG Umesh L. Hepatic involvement in dengue fever in children. Iran J Pediatr. (2012) 22(2):231–236. 23056891 PMC3446077

[23] Khan MAS Al Mosabbir A Raheem E Ahmed A Rouf RR Hasan M. Clinical spectrum and predictors of severity of dengue among children in 2019 outbreak: a multicenter hospital-based study in Bangladesh. BMC Pediatr. (2021) 21(1):478. 10.1186/s12887-021-02947-y 34715835 PMC8555185

[24] Mishra S Ramanathan R Agarwalla SK. Clinical profile of dengue fever in children: a study from Southern Odisha, India. Scientifica (Cairo). (2016) 2016:6391594. 10.1155/2016/6391594 27213083 PMC4860230

[25] Balanza N Erice C Ngai M Varo R Kain KC Bassat Q. Host-based prognostic biomarkers to improve risk stratification and outcome of febrile children in low- and middle-income countries. Front Pediatr. (2020) 8:552083. 10.3389/fped.2020.552083 33072673 PMC7530621

[26] Baqi A Ur Rehman F Memon PS Omair SF. Prevalence and outcomes of myocarditis in dengue-infected patients admitted to a tertiary care hospital of low-middle income country. Glob Heart. (2022) 17(1):44. 10.5334/gh.1129 35837358 PMC9231571

[27] Naderian R Paraandavaji E Maddah AH Keshavarzi S Habibian A Naderian R. Pathophysiology and clinical implications of dengue-associated neurological disorders. Front Microbiol. (2025) 16:1536955. 10.3389/fmicb.2025.1536955 41019525 PMC12463822

[28] Raza MA Khan MA Ejaz K Haider MA Rasheed F. A case of dengue fever with hemorrhagic manifestations. Cureus. (2020) 12(6):e8581. 10.7759/cureus.8581 32670716 PMC7358921

[29] Zhang H Zhou YP Peng HJ Zhang XH Zhou FY Liu ZH. Predictive symptoms and signs of severe dengue disease for patients with dengue fever: a meta-analysis. Biomed Res Int. (2014) 2014:359308. 10.1155/2014/359308 25097856 PMC4100454

[30] Nasir M Irfan J Asif AB Khan QU Anwar H. Complexities of dengue fever: pathogenesis, clinical features and management strategies. Discoveries (Craiova). (2024) 12(2):e189. 10.15190/d.2024.8 40093849 PMC11910338

[31] Wiwanitkit V. Bleeding and other presentations in Thai patients with dengue infection. Clin Appl Thromb Hemost. (2004) 10(4):397–398. 10.1177/107602960401000414 15497028

[32] Tewari K Tewari VV Mehta R. Clinical and hematological profile of patients with dengue fever at a tertiary care hospital - an observational study. Mediterr J Hematol Infect Dis. (2018) 10(1):e2018021. 10.4084/mjhid.2018.021 29531658 PMC5841935

[33] Gupta N Boodman C Jouego CG Van Den Broucke S. Duration of fever in patients with dengue: a systematic review and meta-analysis. Am J Trop Med Hyg. (2024) 111(1):5–10. 10.4269/ajtmh.23-0542 38744269 PMC11229643

[34] L’Azou M Moureau A Sarti E Zambrano NJ Wartel B Villar TA. Symptomatic dengue in children in 10 Asian and Latin American countries. N Engl J Med. (2016) 374(12):1155–1166. 10.1056/NEJMoa1503877 27007959

[35] Sáez-Llorens X Tricou V Yu D Rivera L Jimeno J Villarreal AC. Immunogenicity and safety of one versus two doses of tetravalent dengue vaccine in healthy children aged 2–17 years in Asia and Latin America: 18-month interim data from a phase 2, randomised, placebo-controlled study. Lancet Infect Dis. (2018) 18(2):162–170. 10.1016/S1473-3099(17)30632-1 29122463

[36] James MM Manikandan S Balabaskaran Nina P Balasubramani K Gopalan N Behera SK. Associations between climatic variables and dengue incidence in high-burden countries: a systematic review and meta-analysis. Front Clim. (2026) 8:1804553. 10.3389/fclim.2026.1804553

[37] Ribeiro SP Cruz TC Vieira-Duarte R Monteiro JCL Veloso GA Silva MC. Impact of temperature on the incidence of dengue fever in an endemic urban area. Discover Public Health. (2025) 22(1):816. 10.1186/s12982-025-01218-w

[38] Frentiu FD. Dengue fever: the impact of increasing temperatures and heatwaves. EBioMedicine. (2023) 92:104611. 10.1016/j.ebiom.2023.104611 37182266 PMC10200830

[39] Ye G Xu Z Yang M Wang J Liang J Yin J. Clinical features and transmission risk analysis of dengue virus infections in Shenzhen, during 2014-2019. Comput Struct Biotechnol J. (2023) 21:3728–3735. 10.1016/j.csbj.2023.07.001 37560123 PMC10407296

[40] Malavige GN Ogg GS. Pathogenesis of vascular leak in dengue virus infection. Immunology. (2017) 151(3):261–269. 10.1111/imm.12748 28437586 PMC5461104

[41] Liu Z Zhang Q Li L He J Guo J Wang Z. The effect of temperature on dengue virus transmission by Aedes mosquitoes. Front Cell Infect Microbiol. (2023) 13:1242173. 10.3389/fcimb.2023.1242173 37808907 PMC10552155

[42] Sharp TM Anderson KB Katzelnick LC Clapham H Johansson MA Morrison AC. Knowledge gaps in the epidemiology of severe dengue impede vaccine evaluation. Lancet Infect Dis. (2022) 22(2):e42–e51. 10.1016/s1473-3099(20)30871-9 34265259 PMC11379041

[43] Lin M. Incidence and severity of dengue in pediatric patients higher in Southeast Asia versus Latin America (2025). Available online at: https://www.2minutemedicine.com/incidence-and-severity-of-dengue-in-pediatric-patients-higher-in-southeast-asia-versus-latin-america/ (Accessed March 24, 2016).

