# Supplementary material for: Clinical profile and predictors of adverse outcomes in pediatric dengue fever presenting with febrile illness: a prospective observational study in Burao, Somaliland
Source: Front Pediatr. 2026 Jul 14;14:1891591. doi: 10.3389/fped.2026.1891591 (PMC13407834; doi:10.3389/fped.2026.1891591)
Supplement: Supplementary file 1 [file Table1.docx]

Supplementary Table S1. **Sensitivity analyses using alternative multinomial logistic regression models**

| **Model** | **Alternative specification** | **Rationale** | **Summary of findings** |
| --- | --- | --- | --- |
| Primary | Prespecified causal model | Primary analysis | Reported in Table 3 |
| A | Added rash | Assess robustness to inclusion of an additional clinical predictor | Death estimates unstable owing to sparse data; conclusions unchanged |
| B | Age categorized | Assess effect of alternative age parameterization | Estimates for death unstable because of sparse data; conclusions unchanged |
| C | Added hospitalization | Assess influence of disease management variable | Effect estimates similar to primary model; conclusions unchanged |
